# Prevalence and associated risk factors for syphilis in women with recurrent miscarriages

**Published:** 2014

**Authors:** Arshad Hussain Laghari, Viqar Sultana, Akhtar Hussain Samoo, Pirbhomal Makhija, Jehan Ara, Hira

**Affiliations:** 1 Arshad Hussain Laghari, M.Phil Biochemistry, Assistant Professor, Department of Biochemistry, Ghulam Muhammad Mahar Medical College, Sukkur, Pakistan.; 2 Viqar Sultana, PhD Biochemistry, Professor, Biotechnology and Drug Development Laboratory, Department of Biochemistry, University of Karachi, Karachi – 75270, Pakistan.; 3 Akhtar Hussain Samoo, FCPS Medicine,Assistant Professor, Department of Physiology, Ghulam Muhammad Mahar Medical College, Sukkur, Pakistan.; 4 Pirbhomal Makhija, FCPS Medicine, Senior Registrar, Department of Medicine, Ghulam Muhammad Mahar Medical College, Sukkur, Pakistan.; 5 Jehan Ara. PhD, Professor, Postharvest Technology Laboratory, Department of Food Science and Technology, University of Karachi, Karachi – 75270, Pakistan.; 6 Hira, Lecturer, Biotechnology and Drug Development Laboratory, Department of Biochemistry, University of Karachi, Karachi – 75270, Pakistan.

**Keywords:** Syphilis, Treponema Pallidum infection, Recurrent miscarriages, VDRL, FTA-ABS

## Abstract

***Objective: ***A Cross Sectional population based serological studies was conducted to determine the prevalence and associated risk factors for syphilis women with recurrent miscarriages.

***Methods: ***Patient’s 5ml whole blood was collected through venepuncture technique. Data were collected by all women answered a questionnaire and by investigating blood sample VDRL test and FTA-ABS test. The study was conducted in a confidential manner and numbers were used to identify the participant.

***Results: ***Total 256 women were included in the present study. Mean age of women was 29.4 years while range was 21 to 38 years (206/256). Out of the 256 samples, 05 (1.9%) were positive for active syphilis. Majority belonged to low socioeconomic group, uneducated and had previous congenital anomaly.

***Conclusion: ***Active infection with *Treponema pallidum* (T.P) in women belonging to low socioeconomic level were disquieting. This is probably due to illiteracy and high proportion of unsafe sexual behavior. It is also suggestive that seropositive status is often discovered in routine serological studies during pregnancy.

## INTRODUCTION

As in most resource-limited countries, widespread screening is not conducted in Pakistan for many important infections and metabolic diseases during pregnancy and in newborn, including syphilis, HIV, gonorrhea, Chlamydia and inborn error of metabolism.^1^ In addition, Pakistan has one of the highest fertility rates of any country in the world.^2^ Syphilis in pregnant women is associated with low birth weight, prematurity, and intrauterine death.^3^ Syphilis also has potential association between syphilis seropositivity and miscarriage.^4^

Active syphilis infection in developed countries is rare. But the high magnitude of the problems associated with congenital syphilis is still reported in part of the world where the traditional venereal disease has not been controlled, such as remote areas of Pakistan.^5^

*Treponema pallidum *(TP) is a spirochaete bacterium and the main species of medical importance that causes syphilis. Pathogenic treponemes are found in the lesions of syphilis.5 The treponemes are highly infectious and saprophytic treponemes can be found on mucous membranes in the mouth, genital tract and also in skin ulcers.^6^ Acquired syphilis which is transmitted congenitally or sexually has primary, secondary, or tertiary stages.^7^ In congenital syphilis, an untreated mother with syphilis infects her unborn fetus. The treponemes pass through the placenta in the blood. The fetus is infected with T.P that can complicate early pregnancy.^8^

However, certain risk factors associated with a high prevalence of syphilis include maternal age, husband’s occupation, , late antenatal care, illiteracy, unemployment, habitual drug use, husband’s habitual drug use, husband’s extramarital relation, and unscreened blood transfusion.^8^,^9^

The true burden of syphilis in our country is unknown. The main reasons are that the mothers are mostly asymptomatic or have non-specific symptoms, lack of awareness about such diseases, inadequate facilities for screening tests or their high cost, poor access to a health facility and nonexistence of surveillance systems.^9^^,^^10^

The present study was designed to estimate the prevalence and associated risk factors for syphilis in women with recurrent miscarriages attending Gyn & Obsc Clinic of Ghulam Muhammad Mahar Medical College Teaching Hospital, Sukkur with history of recurrent miscarriages.

## METHODS

This was a cross-sectional study conducted at Gyn & Obs clinic of Ghulam Muhammad Mahar Medical College Teaching Hospital, Sukkur. The woman almost belonged to remote area of Sindh, Pakistan. Most of the women were residents of those areas where people work as laborers, farmers and some do odd jobs selling forest products. Houses are made of mud or bricks and cements without any cement plaster. Most of them did not have clean water supply. Many women were illiterate and even many of them were not properly guided about their basic ethics of their religions.

***Study population*** ***:*** Patients were enrolled after taking their informed consent attending Gyn & Obs clinic of Ghulam Muhammad Mahar Medical College Teaching Hospital, Sukkur with history of recurrent miscarriages. Ghulam Muhammad Mahar Medical College Teaching Hospital, Sukkur provides health care services not only to the people of Sukkur constituency, but also to a large number of patients from other locality of Sindh and Baluchistan province. The study was conduct over a period of 14 months which was stared in May, 2012. 

***Data collection: ***The patients were interviewed by using a structured questionnaire to collect the bio-data and history of patients. General physical examination was made by the help of lady doctor and blood samples were obtained for serological analysis.

***Blood sample:*** Approximately 5ml Blood sample were drawn by using disposable syringe through vein puncture technique from cubital vein. Approximately 2.5 ml blood **was** transferred **into** an aliquot containing EDTA immediately after collection of blood sample for hematological analysis, and remaining blood was transferred into a plain tube for biochemical analysis. Samples were taken to the laboratory as soon as possible. The plain tubes were centrifuged at 3000 rpm for 10 minutes to obtain the serum which was tested for syphilis using both VDRL and FTA-ABS assay. 

***Syphilis serology testing and case definition:*** The serum was tested for Venereal Disease Research Laboratory (VDRL) test and Fluorescent Treponemal Antibody – Absorption (FTA-ABS) assay. The definition of active syphilis (both VDRL and FAT-ABS positive) used in this study was based on the serological finding expected for the various stage of disease. Women who had both a non-reactive VDRL test and a non-reactive FAT-ABS assay were considered to be negative for syphilis. Those who were reactive with VDRL test and non-reactive FTA-ABS were considered to be false-positive. And those who were reactive with FTA-ABS and non-reactive with VDRL were considered as old treated. 

## RESULTS

***Syphilis serological results: ***Total 256 women were enrolled with a history of recurrent miscarriages and tested in this study, Out of 256 women, 05 (1.9%) has serological results that were considered active syphilis infection (VDRL test reactive and reactive with FTA-ABS assay). Women reactive with VDRL only and considered as false-positive or very early infection were 04 (1.6%) (They were reactive with VDRL test but non- reactive with FTA-ABS assay).

***Multivariate analysis:*** Risk factors were included in multivariable analysis based on the strength of association criteria cited in methods above: maternal age, husband’s occupation, an income, late antenatal care, illiteracy, unemployed, habitual drug use, husband’s habitual drug use, husband’s extramarital relation, and blood transfusion. 

**Table–I:** Serological results of syphilis in women with recurrent miscarriage

*Results*	*No. of women* *(n = 256)*	*Interpretation*
VDRL and FTA-ABS Non-reactive	247 (96.5%)	No evidence of syphilis
VDRL reactive and FTA-ABS Non-reactive	04 (1.6%)	False – positive or very early infection
VDRL and FTR-ABS Reactive	05 (1.9%)	Active syphilis

**Table–II:** Prevalence (%) crude prevalence odds ratio (PORs) and corresponding 95% confidence interval (CI) or p-value of syphilis by socio-demographic risk factors in a population of women with recurrent miscarriage

*Socio-demographic risk factors*	*Prevalence (%)*	*POR(95% CI) or p-value* *
Total	**1.9 ( 0.6 – 2.3 )**	
Maternal age ˂ 18 years	**1.2 ( 0.4 – 1.0 )**	**2.4 ( 0.6 – 9.0 )**
No school / primary education only	**1.7 ( 0.5 – 1.1 )**	**2.1 ( 0.4 – 8.1 )**
Unemployed (housewife)	**1.6 ( 0.6 – 1.4 )**	**1.9 ( 0.5 – 11.1)**
Husband with no school/primary education only	**1.4 ( 0.4 – 1.0 )**	**1.7 ( 06 -13.4 )**
Husband self-employed	**1.3 ( 0.5 -1.1 )**	**P = 0.40**
Monthly income (Pak Rupees) ≤ 7000	**0.9 ( 0.4 – 1.3 )**	**( 0.6 – 8.4)**

* Chi-square p-value presented when POR could not be computed due to a cell containing zero

**Table–III:** Prevalence (%) crude prevalence odds ratio (PORs) and corresponding 95% confidence interval (CI) or p-value of syphilis by clinical and behavioral risk factors in a population of women with recurrent miscarriage of rural Sindh, Pakistan

*Clinical and behavioral risk factors*	*Prevalence (%)*	*POR(95% CI) or p-value* *
Gravidity ˃ 1	**1.2 ( 0.6 – 2.1 )**	**2.4 ( 0.2 – 13.4 )**
≥ 1 live children	**1.0 ( 0.5 – 2.4 )**	**1.7 (0.4 – 7.9)**
History of previous low birth weight delivery	**0.9 ( 0.3 – 1.0 )**	**2.1 ( 0.5 – 1.0 )**
History of previous congenital anomaly	**1.7 ( 0.4 – 1.3 )**	**1.9 (0.4 – 6.1 )**
No contraceptive use before current pregnancy	**0.4 ( 0.4 – 1.8 )**	**1.1 ( 0.5 – 4.1 )**
5 + years of marriage	**1.9 ( 0.4 – 5 )**	**2.4 ( 0.6 – 13.1**
Presence of genitourinary symptoms	**2.2 ( 0.6 – 12 )**	**2.1 ( 0.4 – 11.7 )**
Presence of genitourinary symptoms in husband	**1.3 ( 0.4 – 6.1 )**	**1.7 ( 0.5 – 3.4 )**
Habitual drug use	**0.3 (0.3 – 3.1 )**	**P = 0.5**
Husband’s habitual drug use	**1.3 ( 0.6 – 4.8**	**2.4 ( 0.6 – 7.7 )**
History of blood transfusion	**1.7 ( 1.3 – 1.4 )**	**1.1 ( 0.4 – 4.3**

* Chi-square p-value presented when POR could not be computed due to a cell containing zero

## DISCUSSION

The seroprevalence of syphilis observed in the present study was 1.9%. This is significantly high as compared to rate in USA, Mediterranean, Pacific, and in European countries.^11^ A piece of research work among blood donors at Lahore found 0.78% seropositive for syphilis.^12^ Another study conducted in Lahore in men presenting with dermatological complaint, showed 31.6% men positive for syphilis.^13^ Another cross sectional clinic based study conducted in Dhaka, Bangladesh in two urban primary health care level clinics among 1103 women found 1.5% prevalence of syphilis.^14^ A study in Bulgaria shown 0.9% prevalence of syphilis.^15^ But a study from China showed sero prevalence was 0.5% which is very low as compared to this study.^16^ In addition, it was studied that in neighboring India, the prevalence of syphilis ranges between 2.0 – 4.8% among women of reproductive age.^17^

Differences in the prevalence of syphilis according to race and sex have been reported.^18^ It was reported that man 20 – 29 years old had consistently higher rate of infection compared to age matched women, 5.2: 1 ratio was estimated (male to female ratio).^19^^,^^20^

Epidemiologically data on prevalence of syphilis among women in Pakistan are not available, however according to World Bank report the total burden of disease was about 350 Disability Adjusted Life Years per 1000 population per year. Sexually transmitted infection formed 2.2% of Disability Adjusted Life Years. Of which syphilis was 0.50% in Pakistan.^21^

Sexual contact is the primary mode of transmission of syphilis and all the women denied extramarital sex. As the information was collected from women and not from their husbands therefore it was possible that the men might have had risky behavior unknown to their spouses.

Significant association of syphilis with travel of sex partner in past one year such as among long distance truck drivers^22^ and drug abusers has been reported. The risk of transmission through blood was negligible due to improved donor’s serologic testing and of refrigerated blood components.^23^ In this study also blood transfusion history was not significantly related to infection. To increase the percentage of screening for syphilis during pregnancy for women at risk, collaborative efforts would be needed among health care providers, health insurances, policymakers and public.

## CONCLUSION

The findings presented here conclude that the syphilis infection is present in women of reproductive age which requires control and prevention. Pregnant women’s knowledge about the importance of early antenatal care, awareness of risk factors, consultation and promotion of knowledge about infection, antenatal syphilis screening are needed.

## Author’s contribution:

**AHL** conceived, designed the study, performed laboratory analysis and prepared the final manuscript. **VS** contributed in laboratory analysis and involved in manuscript writing.

**AHS, PM** were involved in clinical assessment of patients. **JA** did statistical analysis.

**H** was involved in sample and data collection.
